# Surface Microhardness of Enamel Exposed to Supplemented and Unsupplemented Artificial Saliva Formulations In Vitro

**DOI:** 10.3390/jfb17100528

**Published:** 2026-10-19

**Authors:** Aslı Silkü Oflioğlu, Hülya Erten Can, Sude Eylül Ulucan, Ulaş Baysan, Hanife Altınışık, Shakhrukh Abdukadirov

**Affiliations:** 1 Department of Restorative Dentistry, Faculty of Dentistry, Dokuz Eylül University, Izmir 35340, Türkiye; 2 Science and Technology Application and Research Center, Dokuz Eylül University, Izmir 35390, Türkiye; 3 Department of Gastronomy and Culinary Arts, Faculty of Tourism, Dokuz Eylül University, İzmir 35395, Türkiye; 4 Department of Restorative Dentistry, Faculty of Dentistry, Gazi University, Ankara 06490, Türkiye; 5 Faculty of Dentistry, Khoja Akhmet Yassawi International Kazakh-Turkish University, Turkistan 161200, Kazakhstan

**Keywords:** artificial saliva, olive leaf extract, *Olea europaea*, surface microhardness, tricalcium phosphate, sodium fluoride, calcium glycerophosphate, SEM–EDX

## Abstract

This in vitro study compared enamel surface microhardness responses to sodium fluoride (NaF), tricalcium phosphate (TCP), and calcium glycerophosphate (CaGP) in an olive leaf extract-containing artificial saliva carrier and to the unsupplemented carrier. A total of 131 human premolars were randomly allocated to NaF (*n* = 40), TCP (*n* = 40), CaGP (*n* = 40), or carrier (*n* = 11). After 96 h of demineralization, specimens underwent continuous immersion for 28 days. The primary outcome was surface microhardness recovery (ΔVHN), calculated as post-treatment minus post-demineralization Vickers hardness number (VHN). Scanning electron microscopy with energy-dispersive X-ray spectroscopy (SEM–EDX) was exploratory (*n* = 14). Surface hardness increased in all groups. Mean ΔVHN was 105.00 ± 53.00, 138.11 ± 50.40, 102.81 ± 48.46, and 116.98 ± 39.71 for NaF, TCP, CaGP, and carrier, respectively. The TCP formulation showed greater ΔVHN than the NaF and CaGP formulations (Bonferroni-adjusted *p* = 0.027 and 0.009), but no supplemented formulation showed significantly greater recovery than the carrier after multiplicity correction. Supplementary analysis likewise did not demonstrate a TCP–carrier difference (adjusted *p* = 0.117). No corrected SEM–EDX stage contrast was significant. These results describe surface mechanical responses of specific formulations at unequal doses under prolonged laboratory exposure. They do not establish incremental benefit over the calcium/phosphate-containing carrier or superior remineralizing efficacy of TCP; complete lesion repair cannot be inferred.

## 1. Introduction

Saliva plays a critical role in maintaining oral homeostasis by contributing to lubrication, mastication, swallowing, speech, and protection of oral tissues. In addition to its mechanical functions, saliva provides essential buffering capacity, antimicrobial activity, and a continuous supply of calcium and phosphate ions that support the balance between enamel demineralization and remineralization [1,2].

Reduced salivary flow or altered salivary composition can compromise these protective functions and increase susceptibility to dental caries, mucosal irritation, opportunistic infections, and difficulties in speaking or swallowing. Salivary gland hypofunction and xerostomia may occur in association with systemic conditions, head-and-neck radiotherapy, and the use of various medications [3,4]. Artificial saliva preparations are therefore commonly used to alleviate oral dryness and partially compensate for the functional consequences of reduced salivary secretion [5,6].

Conventional artificial saliva formulations are primarily designed to reproduce the lubricating and moisturizing properties of natural saliva; however, their ability to reproduce the complex protective, buffering, and mineral-regulating functions of natural saliva remains limited. In particular, formulations intended primarily for symptomatic relief may not provide sufficient bioactive components to support enamel mineral balance. Consequently, increasing attention has been directed toward multifunctional artificial saliva systems incorporating remineralizing, antimicrobial, antioxidant, or other biologically active components [7].

Among established remineralizing strategies, fluoride remains widely used because of its ability to reduce enamel demineralization and enhance mineral redeposition and acid resistance [8,9,10]. Calcium-phosphate-based compounds, including tricalcium phosphate (TCP) and calcium glycerophosphate (CaGP), have also been investigated as remineralizing agents capable of supporting mineral recovery in demineralized enamel [11,12].

In parallel, plant-derived bioactive compounds have attracted interest as potential functional components of oral-care formulations. Olive leaf extract (*Olea europaea* L.) contains phenolic compounds, including oleuropein and hydroxytyrosol, and has demonstrated substantial antioxidant activity [13,14]. Its antimicrobial activity has also been reported [13]. Our previously developed olive leaf extract-enriched artificial saliva formulation demonstrated antioxidant activity and favorable cytocompatibility in gingival fibroblast cultures [7]. These findings supported its use as a bioactive carrier system for further investigation. However, the independent effect of olive leaf extract on enamel mineral recovery has not been established, and its presence within an artificial saliva formulation should not itself be interpreted as evidence of a remineralizing effect.

Evidence on surface mechanical responses to supplemented artificial saliva formulations remains limited. Comparisons within a common carrier can describe differences among specific formulations, but a calcium/phosphate-containing carrier is an active reference rather than an ion-free negative control. Such a design cannot isolate the independent effect of olive leaf extract or establish restoration of lesion mineral content from surface hardness alone.

The aim of this in vitro study was to compare enamel surface microhardness recovery after exposure to an unsupplemented olive leaf extract-containing artificial saliva carrier and to the same carrier supplemented separately with NaF, TCP, or CaGP at the specified concentrations. SEM–EDX was used for exploratory assessment of surface calcium (Ca), phosphorus (P), and Ca/P weight ratio. The comparison concerned complete formulations rather than dose-equivalent tests of individual agents.

The null hypothesis was that supplementation of the olive leaf extract-enriched artificial saliva carrier with NaF, TCP, or CaGP would not result in significant differences in enamel surface microhardness recovery among the tested formulations.

## 2. Materials and Methods

### 2.1. Study Design

This in vitro experimental study was conducted to compare the effects of sodium fluoride (NaF)-, tricalcium phosphate (TCP)-, and calcium glycerophosphate (CaGP)-supplemented olive leaf extract-enriched artificial saliva formulations on the surface microhardness and elemental composition of experimentally demineralized human enamel. Vickers surface microhardness was used as the primary quantitative outcome, while SEM–EDX analysis was performed on a subset of specimens to assess surface morphological and elemental changes.

The study was conducted at Dokuz Eylül University Faculty of Dentistry and collaborating laboratories within the university. Ethical approval was obtained from the Research Ethics Committee of the Faculty of Dentistry, Dokuz Eylül University (Approval No. 2026/29-20). The ethical approval covered the collection and experimental use of extracted human teeth. All teeth had been extracted exclusively for clinical indications, and no tooth was removed specifically for research purposes. Written informed consent was obtained for the use of the extracted teeth for research purposes.

A total of 131 human permanent premolars were included. One enamel specimen was obtained from each tooth; therefore, each specimen represented an independent experimental unit. A subset of 14 specimens was additionally evaluated by SEM–EDX. The study was reported with reference to the Checklist for Reporting In Vitro Studies (CRIS) recommendations to improve methodological transparency and reproducibility [15].

### 2.2. Tooth Sample Preparation and Group Allocation

Extracted sound human permanent premolars with intact enamel surfaces were included. Teeth presenting caries, restorations, cracks, developmental enamel defects, or other structural alterations were excluded.

Following extraction, residual soft-tissue remnants were removed and the teeth were disinfected and stored in 0.2% thymol solution at 4 °C for less than 2 months until specimen preparation. The storage period was limited to less than 2 months because previous evidence indicates that short-term storage of extracted teeth in thymol-containing media preserves enamel microhardness within an acceptable range for in vitro mechanical testing, whereas substantially longer storage may alter microhardness [16]. From each tooth, one enamel specimen measuring approximately 3 × 3 mm was prepared using a low-speed diamond saw under continuous water cooling. The enamel surfaces were polished to obtain flat and standardized surfaces suitable for surface microhardness measurements.

Before baseline surface microhardness measurements, eligible specimens were randomly allocated to the four formulation groups using computer-generated random numbers. The final allocation was NaF (*n* = 40), TCP (*n* = 40), CaGP (*n* = 40), and carrier control (*n* = 11). The available sample consisted of teeth extracted exclusively for clinical indications. Equal numbers were assigned to the three supplemented formulations, which were the principal formulation comparisons, while the remaining 11 eligible specimens formed the carrier-control group. The unequal allocation reduces the precision of comparisons involving the carrier control.

Prospective planning and the reported sensitivity analysis. A sample-size calculation was undertaken prospectively, before experimentation. The quantitative assessment reported below is a separate sensitivity analysis using the final group sizes; it does not report the original prospective design assumptions or provide an a priori justification of the 40:40:40:11 allocation.

Sensitivity analysis of the final allocation. A sensitivity analysis was conducted using the actual group sizes, a two-sided per-comparison α of 0.05/6 = 0.00833, and a target power of 80%. Under an illustrative normal-theory independent two-sample *t*-test model with equal population variances, the minimum detectable standardized mean difference was Cohen’s d = 0.796 for 40 versus 40 specimens and d = 1.229 for 40 versus 11 specimens. These thresholds describe detectable effects under the assumed model; they are neither prospective sample-size estimates nor achieved power estimates for the Kruskal–Wallis, Mann–Whitney, or ANCOVA analyses, and do not establish that the sample was adequate for smaller effects. The SEM–EDX subset was exploratory and was not justified by this Vickers sensitivity calculation.

### 2.3. Demineralization Procedure

Baseline surface microhardness measurements were obtained from all specimens before experimental demineralization.

Enamel demineralization was induced using a solution prepared by dissolving 0.6 g calcium carbonate (CaCO_3_) and 1.5 g acetic acid (CH_3_COOH) in 500 mL of distilled water. The pH of the solution was measured and adjusted to 4.4. The selection of pH 4.4 and a 96 h exposure period was consistent with established in vitro enamel demineralization protocols in which artificial enamel lesions were produced at pH 4.4 and 37 °C over 96 h, with regular renewal of the demineralizing medium [17,18]. All specimens were immersed together in the demineralizing solution and maintained at 37 °C in an incubator for 96 h. The demineralizing solution was completely renewed every 24 h throughout the exposure period.

Following demineralization, the specimens were rinsed with distilled water and subjected to a second surface microhardness measurement. Formation of experimentally demineralized enamel was confirmed by the reduction in Vickers surface microhardness relative to baseline values. Because lesion depth was not directly quantified in the present study, no specific subsurface lesion depth was assumed.

### 2.4. Artificial Saliva and Experimental Formulations

The artificial saliva carrier was based on the olive leaf extract-containing formulation previously developed and evaluated by our research group [7]. Table 1 reports the carrier constituents, their nominal preparation concentrations, and the commercial extract volume added to the carrier.

The artificial saliva carrier contained KH_2_PO_4_ (0.33 g/L), Na_2_HPO_4_ (0.34 g/L), KCl (1.27 g/L), NaSCN (0.16 g/L), NaCl (0.58 g/L), CaCl_2_ (0.17 g/L), NH_4_Cl (0.16 g/L), urea (0.20 g/L), glucose (0.03 g/L), ascorbic acid (0.002 g/L), and sodium carboxymethylcellulose (CMC; 10 g/L), together with a commercially available olive leaf extract (*Olea europaea* L.; Kalextract, Balıkesir, Türkiye). Anhydrous CaCl_2_ (Supelco/Merck, product 1.02378.0500; formula mass 110.98 g/mol) and anhydrous Na_2_HPO_4_ (Sigma-Aldrich, ReagentPlus, product S0876-100G; formula mass 141.96 g/mol) were used.

Commercial liquid olive leaf extract (Kalextract, Balıkesir, Türkiye) was added at 0.5 mL per 1000 mL of artificial saliva carrier in all four formulations. This corresponds to a nominal loading of 0.05% (*v*/*v*) relative to the carrier volume, rather than a concentration of dry extract or individual phytochemical constituents. The extract loading was therefore not a between-group experimental variable. Batch-specific phytochemical characterization and dry-matter determination were not performed. Manufacturer identification and the disclosed volume loading do not establish batch-specific chemical composition or exclude interactions with the supplements.

For the experimental formulations, NaF, TCP, or CaGP was incorporated separately into the same carrier. For NaF, 0.18 mL of a 20 mg/mL NaF solution (Sigma-Aldrich, St. Louis, MO, USA) was added per liter, giving 3.6 mg/L NaF (approximately 1.63 mg F^−^/L). TCP and CaGP were each added at 2.48 g/L. The unsupplemented carrier contained no added NaF, TCP, or CaGP but retained its calcium- and phosphate-containing salts and olive leaf extract. The experiment did not compare equivalent doses or equivalent calcium, phosphate, or fluoride ion availability. Neither equimolar dosing nor matching of dissolved or freely available ions was performed; nominal mass concentrations do not establish soluble-ion equivalence. Accordingly, each treatment denotes a complete carrier-plus-supplement formulation at its stated preparation dose. Group contrasts compare these formulations within the shared carrier, rather than isolated compounds with matched ion availability.

### 2.5. Experimental Groups

Specimens had been randomly allocated to the four groups before baseline Vickers measurement. Following demineralization, each specimen received the formulation corresponding to its assigned group:

Group 1—NaF (*n* = 40): olive leaf extract-enriched artificial saliva supplemented with 3.6 mg/L NaF.

Group 2—TCP (*n* = 40): olive leaf extract-enriched artificial saliva supplemented with 2.48 g/L TCP.

Group 3—CaGP (*n* = 40): olive leaf extract-enriched artificial saliva supplemented with 2.48 g/L CaGP.

Group 4—Carrier control (*n* = 11): the same olive leaf extract-containing artificial saliva without added NaF, TCP, or CaGP. This carrier retained its calcium- and phosphate-containing salts.

Because olive leaf extract was present at the same concentration in all four formulations, the experimental design was not intended to determine the independent effect of olive leaf extract on enamel surface microhardness recovery or elemental changes.

### 2.6. Experimental Treatment Protocol

Following demineralization, specimens were continuously immersed in their assigned formulations for 28 days at 37 °C, with 1 L per group. Formulations were freshly prepared and used immediately; fresh replacement formulations were prepared each day and the medium was completely replaced every 24 h. The pH of the initial and freshly prepared replacement formulations was checked and adjusted to 6.82 before use. This value was the preparation target, not a documented constant throughout immersion. No daily numerical pH log was retained, and pH in the spent medium at the end of each 24 h interval was not documented. Consequently, within-interval pH drift cannot be assessed from the available records. Daily preparation and replacement did not establish constant pH during any 24 h immersion interval.

At the end of the 28-day experimental period, the specimens were removed from the solutions, rinsed with distilled water, and subjected to post-treatment Vickers surface microhardness measurements. Specimens selected for SEM–EDX analysis were additionally evaluated for surface morphological and elemental changes.

The 28-day treatment period was selected following a review of published in vitro remineralization studies, providing an extended laboratory observation window under sustained formulation contact. Selection of this duration does not validate continuous immersion as a clinical application schedule or establish an optimal treatment duration. No intermediate treatment-phase measurements were made, so response kinetics were not determined. The static model omits salivary clearance, acquired pellicle and biofilm formation, dietary challenges, and alternating demineralization and remineralization. Daily preparation, pH adjustment and replacement standardized fresh-medium exposure but did not establish physicochemical stability, pH constancy over each 24 h interval, or equivalent soluble-ion availability.

### 2.7. Vickers Surface Microhardness Analysis

Surface microhardness was evaluated using a Vickers microhardness tester (HMV-700 Micro Hardness Tester, Shimadzu Corp., Kyoto, Japan). At each measurement stage, three indentations were made within the central polished enamel area using a diamond indenter under a load of 200 gf for 10 s. Adjacent indentations were separated by a minimum distance of 0.5 mm to prevent interaction between neighboring indentation fields, consistent with previously described enamel microhardness methodology [19]. At subsequent measurement stages, new measurement fields were selected adjacent to, but not overlapping with, the previous indentation sites, and specimen margins were avoided. The three readings obtained at each stage were averaged to obtain one Vickers hardness number (VHN) for each specimen. Measurement fields were standardized using a predefined map to maintain the same approximate anatomical region across stages while avoiding overlap with previous indentations. Intra-operator repeatability was assessed, and instrument calibration was verified during the study.

**Figure 1:**
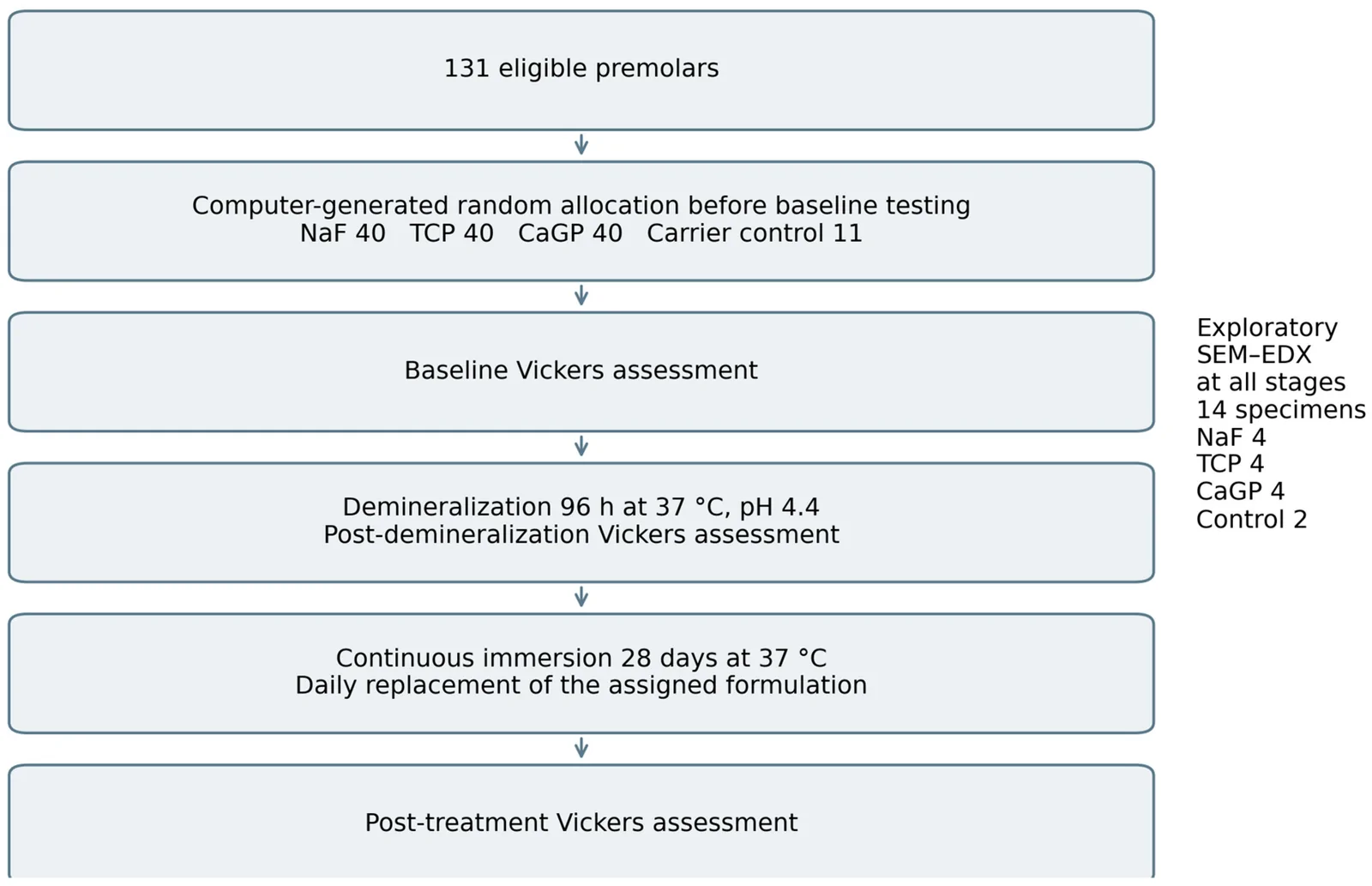
Experimental workflow. Computer-generated random allocation to the four groups occurred before baseline Vickers testing. A total of 131 independent tooth specimens underwent baseline measurement, 96 h demineralization, post-demineralization measurement, and 28-day continuous exposure followed by post-treatment measurement. SEM–EDX was performed on a subset of 14 specimens across the three stages.

Measurements were performed at baseline, after the 96 h demineralization period, and after the 28-day experimental treatment period. The same trained operator performed all surface microhardness measurements and was blinded to the experimental group allocation during Vickers assessment. Surface microhardness recovery during the treatment phase (ΔVHN) was calculated as post-treatment VHN minus post-demineralization VHN. ΔVHN was interpreted as a measure of surface microhardness recovery rather than as evidence of complete structural or subsurface enamel remineralization (Figure 1).

### 2.8. SEM–EDX Analysis

SEM coupled with EDX was used as a complementary exploratory method to evaluate enamel surface morphology and elemental composition. A total of 14 specimens were examined, distributed as follows: NaF (*n* = 4), TCP (*n* = 4), CaGP (*n* = 4), and carrier control (*n* = 2). Specimens were examined using a field-emission scanning electron microscope (ZEISS GeminiSEM 560, Carl Zeiss, Oberkochen, Germany) operated at an accelerating voltage of 12 kV. At each measurement stage, specimens were dried for 24 h before gold–palladium coating. Coating was repeated at each stage, and an acetone treatment was used for coating removal between stages.

SEM images were obtained at baseline, after demineralization, and after the 28-day experimental treatment period. Elemental composition was assessed using the integrated EDX system. Calcium (Ca) and phosphorus (P) were quantified as weight percentages (wt%), and the Ca/P ratio was calculated for each specimen at each experimental stage. The specimen was considered the experimental unit for elemental analysis. Because SEM–EDX was performed on a limited subset, these findings were interpreted as exploratory, descriptive surface-level observations. Surface elemental composition does not establish restoration of subsurface mineral content, identify a deposited mineral phase, or demonstrate complete lesion repair or between-group superiority.

### 2.9. Statistical Analysis

Statistical analyses were performed using IBM SPSS 30.0 Statistics (IBM Corp., Armonk, NY, USA). The distribution of continuous variables was assessed using the Shapiro–Wilk test. Because the unadjusted Vickers data did not consistently meet the assumptions of normality, non-parametric statistical methods were used for the unadjusted comparisons. Continuous data are presented as mean ± standard deviation (SD) and median with interquartile range [IQR], as appropriate. Revision analyses and graphical summaries were independently reproduced in Python 3.10.0 using NumPy and SciPy. For individual estimates using heteroscedasticity-consistent covariance estimator type 3 (HC3) and contrasts, confidence intervals and *p* values used the asymptotic normal reference distribution; the overall HC3 group test was reported as an F test with 3 and 126 degrees of freedom.

Baseline and post-demineralization VHN were compared among groups using the Kruskal–Wallis test. A nonsignificant comparison was not interpreted as demonstrating equivalence. ΔVHN was prespecified as the primary outcome before the experiment. Surface microhardness recovery was calculated as ΔVHN = post-treatment VHN − post-demineralization VHN. Within-group post-demineralization to post-treatment comparisons were secondary analyses using the two-sided Wilcoxon signed-rank test, with Bonferroni correction across the four groups in this revision.

Intergroup differences in ΔVHN were evaluated using the Kruskal–Wallis test. When a significant overall difference was detected, pairwise comparisons were performed using the Mann–Whitney U test with Bonferroni adjustment for the six possible pairwise comparisons. Effect size for the overall Kruskal–Wallis comparison was expressed as epsilon-squared (ε^2^), while rank-biserial correlations were calculated for the significant pairwise comparisons. In the revision, differences in mean ΔVHN were also estimated with specimen-level nonparametric percentile bootstrap confidence intervals using 100,000 resamples within each group (random seed 4609865). Ordinary 95% confidence intervals and Bonferroni intervals for six contrasts (individual coverage 99.1667%, nominal family coverage at least 95%) are provided in Supplementary Table S3. These intervals estimate mean differences and are complementary to the rank-based Mann–Whitney tests; they are not confidence intervals obtained by inverting those rank tests.

Analysis of covariance (ANCOVA) was not prespecified and was subsequently added as a supplementary analysis to account for possible differences in surface microhardness at the beginning of the treatment phase. Post-treatment VHN was entered as the dependent variable, experimental group as the fixed factor, and post-demineralization VHN as the covariate. The homogeneity-of-regression-slopes assumption was assessed by testing the group × post-demineralization VHN interaction and was not violated (*p* = 0.223). Heteroscedasticity-robust HC3 standard errors were used to improve robustness to unequal group sizes and variance heterogeneity. Adjusted post-treatment means with 95% confidence intervals (CIs) were estimated for each group, and pairwise group comparisons were performed with Bonferroni correction. Original baseline VHN was not included as a covariate. Therefore, this model adjusts for measured hardness immediately before treatment, not directly for the significant original baseline imbalance. It cannot establish equivalence of specimens, eliminate unmeasured differences, or convert this supplementary analysis into confirmatory evidence. The prespecified ΔVHN analysis remained the primary analysis. Within-group increases describe changes during exposure and do not demonstrate a supplement effect over the carrier. The analyses address different quantities: rank-based tests compare the distribution of ΔVHN, whereas ANCOVA estimates conditional post-treatment mean VHN at a common measured post-demineralization VHN. Neither replaces the other, and directionally similar results are not independent confirmation. Covariate-adjusted pairwise contrasts are additionally reported with ordinary 95% and Bonferroni family-adjusted confidence intervals in Supplementary Table S4.

**Figure 2:**
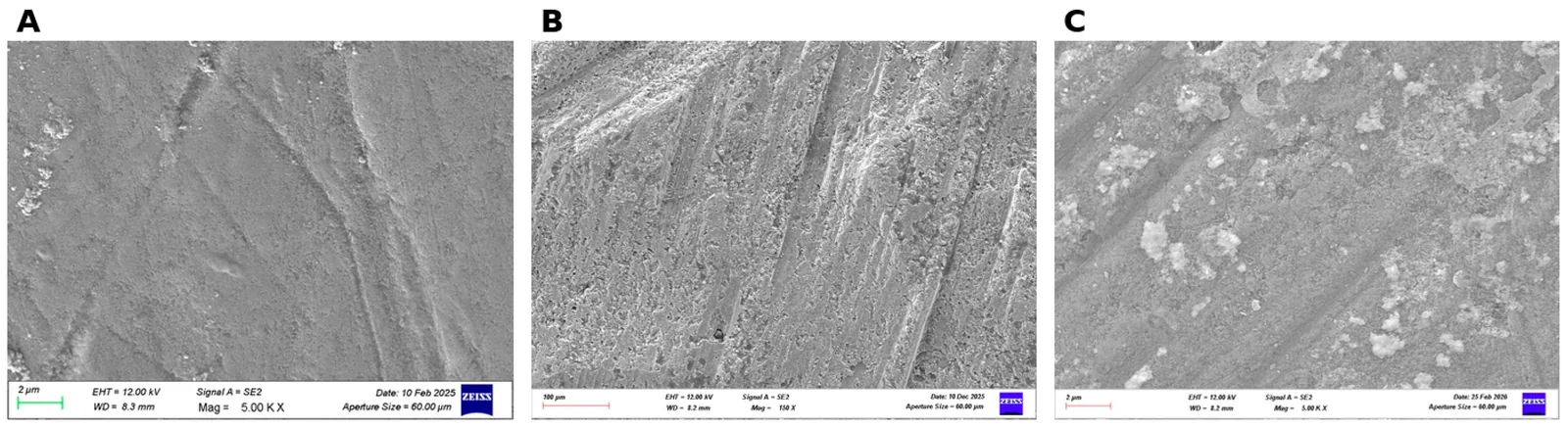
Representative SEM micrographs illustrating enamel surface morphology at (**A**) baseline (5.00 kX; scale bar, 2 μm), (**B**) after experimental demineralization (150×; scale bar, 100 μm), and (**C**) after the 28-day experimental treatment period (5.00 kX; scale bar, 2 μm). The images were acquired at different magnifications and are therefore presented for qualitative illustration only, not for direct quantitative morphological comparison between stages or groups.

SEM–EDX analyses were exploratory. For the supplemented groups, Friedman statistics were evaluated with exact within-specimen stage-label permutations (6^4^ = 1296 assignments per outcome) because each group contained only four specimens. These revision calculations replace the previous chi-square approximation. Stage-specific Wilcoxon signed-rank comparisons used Bonferroni correction across the three stage contrasts for each outcome. The nine omnibus SEM–EDX outcomes were not treated as confirmatory tests, and no inferential between-group elemental comparisons were performed. The two carrier-control specimens were summarized descriptively. All tests were two-sided, and the nominal significance threshold was 0.05 (Figure 2).

## 3. Results

### 3.1. Vickers Surface Microhardness

**Figure 3:**
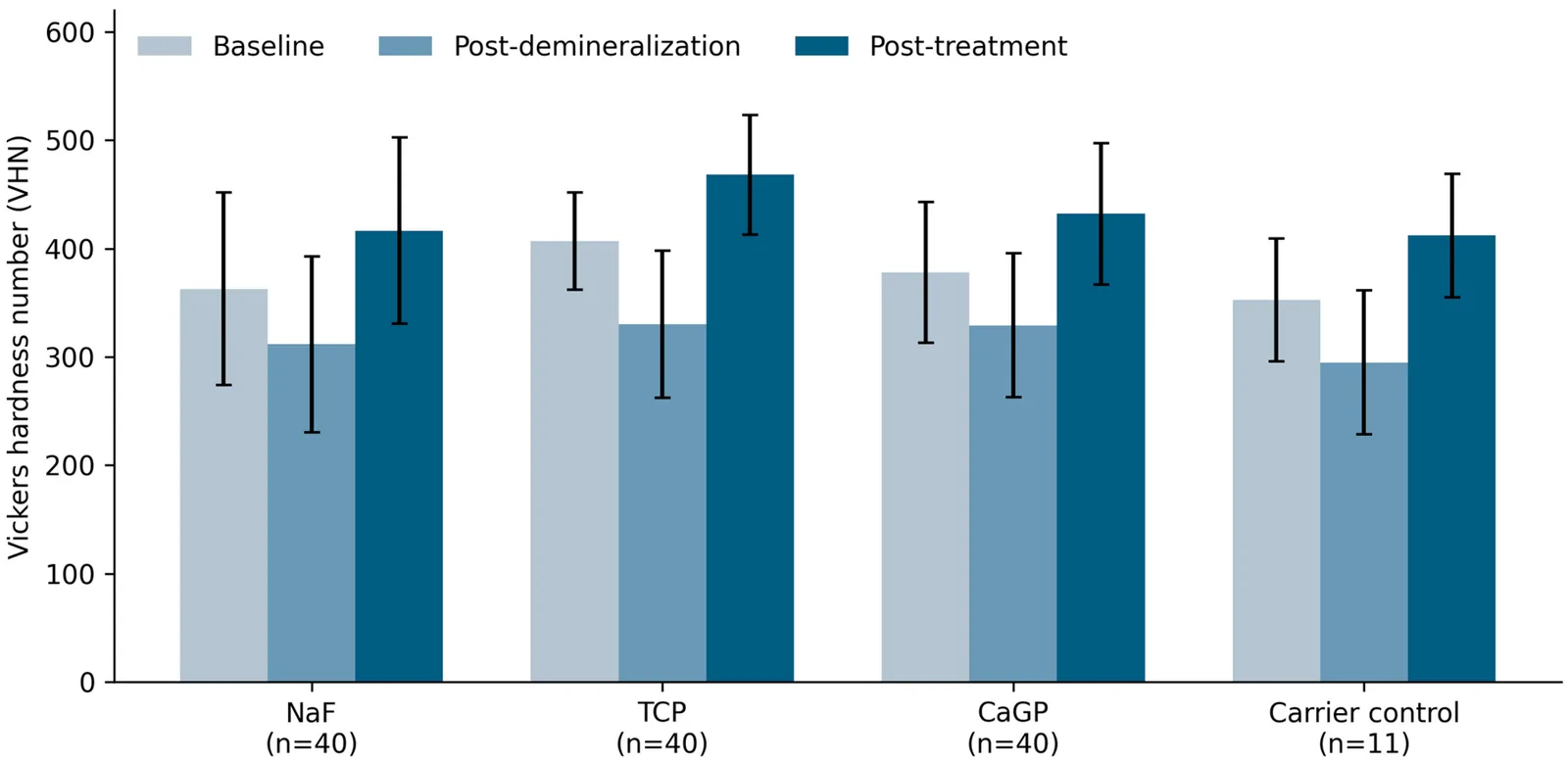
Vickers surface microhardness at baseline, after demineralization, and after 28-day treatment. Bars are means and error bars are SD. Numerical summaries are available in Supplementary Table S1. Within-group Bonferroni-adjusted *p* values are <0.001 for the supplemented groups and 0.004 for the carrier control.

Surface microhardness values across stages are illustrated in Figure 3; numerical summaries are provided in Supplementary Table S1. Baseline VHN differed among groups (Kruskal–Wallis H = 12.712, *p* = 0.005). No statistically significant group difference was detected after demineralization (H = 3.514, *p* = 0.319). This result does not establish equivalence of the groups at the start of treatment. Random allocation preceded baseline testing and did not guarantee balance in this heterogeneous extracted-tooth sample. Chance imbalance is a possible explanation, but the observed baseline difference cannot be assigned a definitive cause from the available data.

Following the 28-day experimental treatment period, surface microhardness increased significantly relative to the post-demineralization condition in all four groups (four-test Bonferroni-adjusted Wilcoxon *p* < 0.001 for the supplemented groups and *p* = 0.004 for the carrier control). Mean ΔVHN values were 105.00 ± 53.00 for NaF, 138.11 ± 50.40 for TCP, 102.81 ± 48.46 for CaGP, and 116.98 ± 39.71 for the carrier control. The overall intergroup difference in ΔVHN was statistically significant (Kruskal–Wallis H = 12.864, *p* = 0.005; ε^2^ = 0.078). Bonferroni-adjusted pairwise Mann–Whitney U comparisons demonstrated significantly greater ΔVHN in the TCP group than in the NaF group (adjusted *p* = 0.027; rank-biserial correlation = 0.369) and the CaGP group (adjusted *p* = 0.009; rank-biserial correlation = 0.414). No statistically significant differences were detected for the remaining pairwise comparisons after Bonferroni adjustment (adjusted *p* > 0.05). These comparisons concern formulations at the tested doses; none showed significantly greater ΔVHN than the carrier after multiplicity correction.

After adjustment for post-demineralization VHN, a significant overall group effect on post-treatment surface microhardness was observed (F(3, 126) = 6.070, *p* < 0.001). Adjusted post-treatment VHN values were 423.56 (95% CI: 407.17–439.95) for NaF, 461.82 (95% CI: 448.12–475.53) for TCP, 426.27 (95% CI: 412.03–440.51) for CaGP, and 430.93 (95% CI: 409.57–452.28) for the carrier control. Bonferroni-adjusted pairwise comparisons showed significantly higher adjusted post-treatment VHN in the TCP group than in the NaF (adjusted *p* = 0.002) and CaGP groups (adjusted *p* = 0.002). No statistically significant difference was detected between TCP and the carrier control (adjusted *p* = 0.117), and the remaining pairwise comparisons were also nonsignificant. The supplementary ANCOVA compares post-treatment VHN conditional on measured post-demineralization VHN. It does not eliminate the original baseline imbalance or other unmeasured differences between extracted teeth. Its directionally consistent estimates are not independent confirmation of the primary ΔVHN findings or evidence of remineralization efficacy.

**Figure 4:**
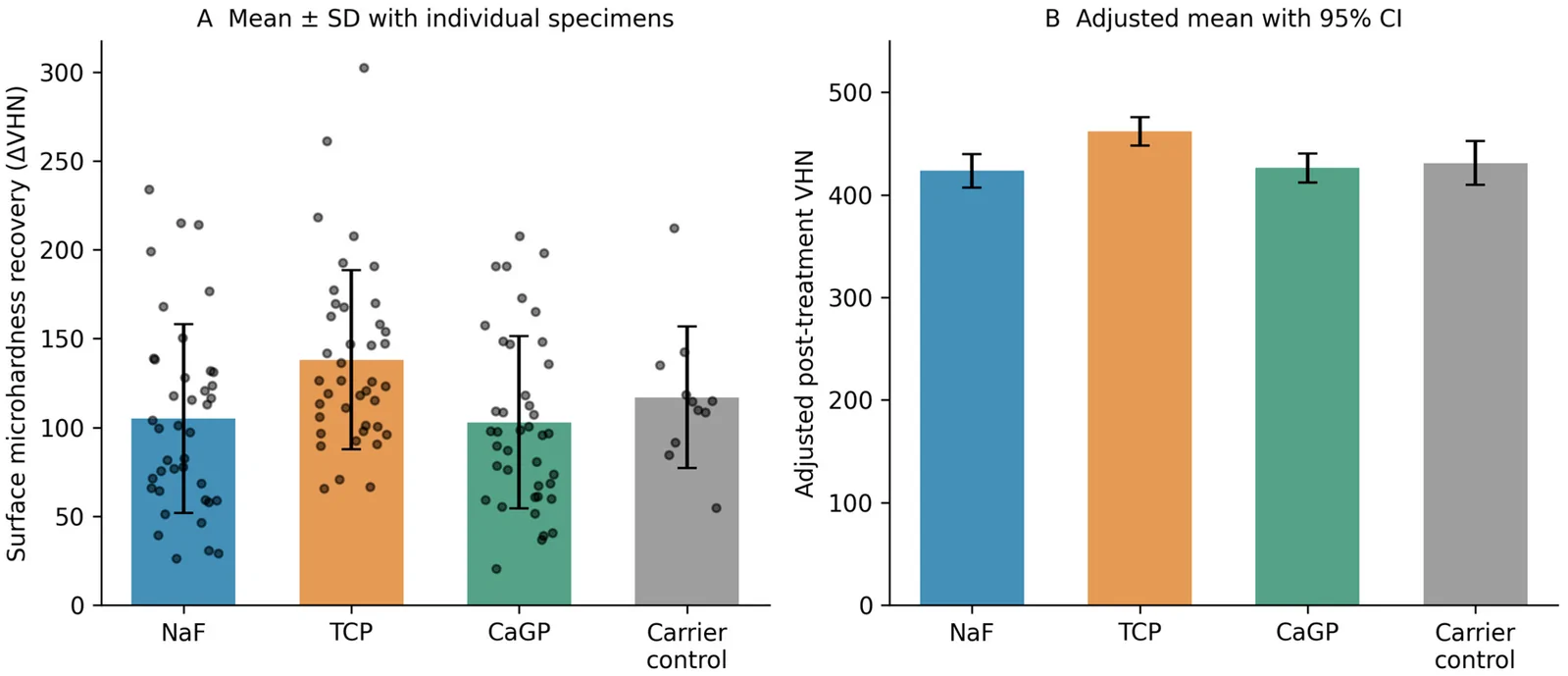
Surface microhardness recovery and adjusted post-treatment VHN. (**A**) Bars show mean ΔVHN with SD and individual tooth specimens. (**B**) Bars show estimated post-treatment means adjusted for post-demineralization VHN with ordinary 95% confidence intervals. Group-mean intervals do not replace multiplicity-adjusted pairwise tests. Numerical values and contrast intervals are provided in Supplementary Tables S2–S4.

Figure 4 summarizes ΔVHN and adjusted post-treatment VHN. The mean ΔVHN difference was 33.11 VHN for TCP minus NaF (bootstrap 95% CI: 11.08–55.59) and 35.30 VHN for TCP minus CaGP (95% CI: 14.18–56.91). Bonferroni family-adjusted bootstrap intervals were 3.35–63.30 and 6.68–64.66 VHN, respectively. For TCP minus the carrier control, the adjusted post-treatment difference was 30.90 VHN; its ordinary 95% CI was 4.97–56.83, while the interval accounting for all six comparisons was −4.00–65.80. The multiplicity-adjusted interval includes zero, consistent with the adjusted *p* value of 0.117. Nonsignificance does not demonstrate equivalence.

### 3.2. SEM–EDX Analysis

**Figure 5:**
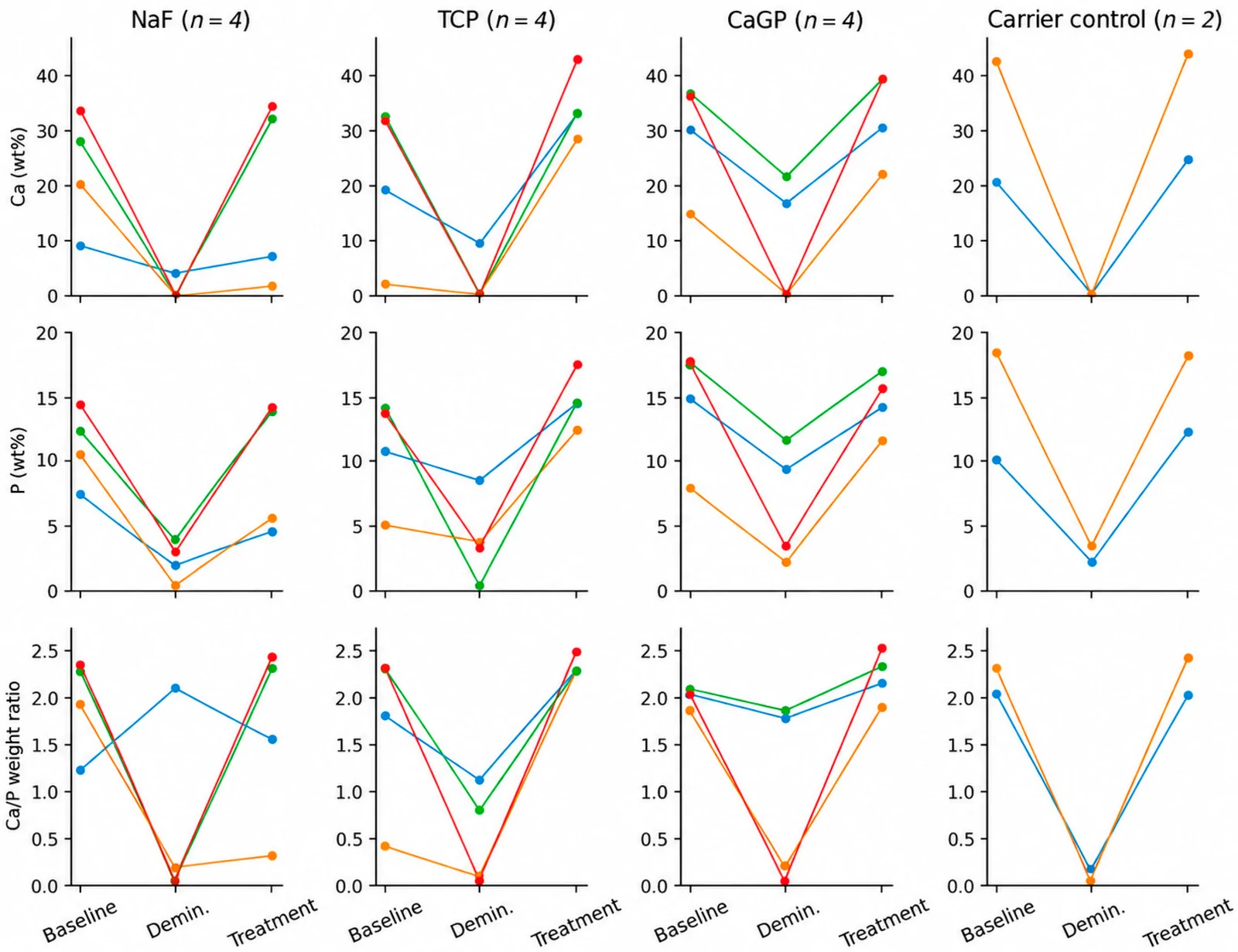
Individual SEM–EDX specimen trajectories for Ca, P, and Ca/P weight ratio. Each colored line connects the three stages of one specimen within a formulation. Supplemented groups contain four specimens each; the carrier control contains two. Axes use common limits within each outcome row. Trajectories are descriptive and do not establish significant stage-specific differences.

Exact permutation Friedman tests suggested overall stage effects for *p* in the NaF group (*p* = 0.0417) and for Ca, P, and Ca/P ratio in the TCP and CaGP groups (*p* = 0.0046–0.0417). The NaF Ca result was not significant using the exact test (*p* = 0.0694; previously *p* = 0.0498 using the chi-square approximation). No Bonferroni-adjusted individual stage-to-stage comparison was significant (all adjusted *p* ≥ 0.375). These exploratory omnibus results do not identify a specific stage contrast or establish mineral recovery. All stage-wise Ca, P, and Ca/P observations are descriptive measurements of surface elemental composition. Table 2 provides descriptive summaries and exact omnibus *p* values; Figure 5 shows the individual specimen trajectories. The carrier control (*n* = 2) is descriptive only (Table 2).

Representative SEM micrographs are provided as qualitative illustrations of surface morphology across the experimental stages. Because the available images were acquired at different magnifications, they were not used for direct quantitative morphological comparison between stages or groups.

## 4. Discussion

This study describes surface mechanical responses to four specific artificial saliva formulations during continuous 28-day exposure. Surface microhardness increased in all groups, including the unsupplemented carrier. The TCP-supplemented formulation had greater ΔVHN than the NaF- and CaGP-supplemented formulations, but none of the supplemented formulations demonstrated significantly greater ΔVHN than the carrier after correction for six comparisons. Rejection of the overall null hypothesis therefore indicates differences among formulations; it does not establish that supplementation improves upon the carrier or that TCP has superior remineralizing efficacy. The carrier is an active reference for the formulation comparison. The absence of an ion-free comparator and of an otherwise matched extract-free carrier prevents separation of background mineral-medium effects, extract effects, and supplement–carrier interactions. SEM–EDX provides exploratory surface observations, not confirmatory mineral-recovery evidence.

The greater surface microhardness recovery observed with TCP compared with NaF and CaGP is consistent with previous studies demonstrating that TCP-containing systems can improve surface hardness and support mineral recovery in experimentally demineralized enamel [11,20]. However, direct comparison with previous studies requires caution because TCP has frequently been investigated in combination with fluoride or in formulations that differ substantially in composition, pH, exposure time, and experimental model. In the present study, TCP was incorporated into an artificial saliva carrier that itself contained calcium- and phosphate-containing components. Calcium-phosphate availability may have contributed to the observed surface response; nevertheless, soluble ion concentrations, ion release, precipitation, and apatite formation were not directly measured. Therefore, the greater ΔVHN observed for TCP should be interpreted as an experimental finding rather than as direct evidence of a specific mineralization mechanism. Karlinsey et al. evaluated a surfactant-modified β-TCP material combined with 5000 ppm fluoride under pH-cycling conditions and reported enhanced hardness recovery compared with fluoride alone [21]. That chemically modified combination differs from the TCP supplementation tested here and cannot establish the same mechanism or effect for the present carrier.

The NaF-containing formulation also produced a significant increase in surface microhardness following treatment. Fluoride is well established for its role in reducing enamel mineral loss and facilitating mineral recovery under appropriate ionic conditions [8,9]. In the present model, however, ΔVHN was significantly lower in the NaF group than in the TCP group. This difference may reflect the distinct composition and behavior of the tested formulations under the present experimental conditions rather than an inherent general superiority of one remineralizing strategy over another. In particular, the relatively low fluoride concentration used in the present artificial saliva formulation, together with the continuous-immersion model and the calcium- and phosphate-containing carrier, should be considered when comparing these findings with studies using conventional fluoride products. Lippert and Juthani showed that fluoride response and the relationship between hardness and mineral loss depended on the artificial lesion protocol in a pH-cycling experiment [22]. This dependence reinforces the need to interpret the present comparison within its fluoride concentration, lesion preparation and exposure conditions.

The CaGP formulation likewise demonstrated significant surface microhardness recovery. Previous studies have reported favorable enamel responses when CaGP was incorporated into fluoride-containing oral-care formulations [12]. Because such studies frequently evaluate CaGP in combination with other active agents, they do not provide a direct equivalent to the present CaGP formulation. In the current experiment, CaGP produced a lower ΔVHN than TCP, while no significant difference was detected between CaGP and NaF. These findings indicate that the response of CaGP within the present artificial saliva carrier differed from that of TCP, but the underlying physicochemical basis cannot be established from the current data because dissolved calcium and phosphate concentrations and possible precipitation phenomena were not evaluated. In an in situ study, Zaze et al. found improved enamel hardness recovery when CaGP was added to low-fluoride toothpastes [23]. These results concern combined fluoride–CaGP formulations and do not establish the efficacy of CaGP alone in the present carrier.

SEM–EDX has been used in previous in vitro studies to assess surface Ca and P [24]. Here, exact permutation omnibus tests suggested variation across stages for some outcomes in the supplemented groups, but no subsequent Bonferroni-adjusted stage-specific contrast was significant (all adjusted *p* ≥ 0.375). In particular, NaF Ca was not significant with the exact omnibus calculation (*p* = 0.0694). These analyses were based on four specimens per supplemented group and were exploratory. No inferential between-group elemental comparison was performed. Any apparent stage-wise changes in Ca, P, or Ca/P are descriptive surface-level findings, not demonstrated elemental recovery. These surface measurements cannot establish restoration of subsurface mineral content, identify a deposited mineral phase, or demonstrate complete lesion repair or superiority of any formulation.

Vickers microhardness is a surface mechanical outcome rather than a direct measure of lesion mineral mass or original enamel architecture. Mean post-treatment VHN exceeded mean baseline VHN in every group, including the carrier. This overshoot is central to interpretation: greater hardness may reflect a deposited mineral-rich surface layer, altered surface composition, or other changes affecting indentation rather than restoration of the original lesion. The experiment did not distinguish these possibilities or identify the deposited phase. A 28-day treatment interval has also been used in a pH-cycling study [25], but that exposure protocol differs from the continuous immersion used here. Lippert and Lynch found that surface hardness changes plateaued while microradiographic lesion depth and integrated mineral loss continued to increase [26]. Accordingly, neither higher ΔVHN nor exploratory surface Ca/P measurements establish more complete lesion repair. Cross-sectional mineral measurements and assessment of surface deposits are required to resolve this distinction.

Olive leaf extract is a common component of all four formulations. Its phenolic constituents have reported antioxidant and antimicrobial activity [13], and the earlier carrier study reported antioxidant activity and favorable cytocompatibility [7]. These observations do not establish an independent enamel-repair effect. Benavente-García et al. characterized olive leaf phenolics and their radical-scavenging activities [27]; those findings neither characterize the batch used here nor demonstrate mineral recovery. The commercial extract volume loading is now disclosed, but its batch-specific phytochemical composition and dry-matter content were not determined. This limits chemically identical reproduction and assessment of possible interactions with the added salts. The shared extract loading enables a within-study comparison but does not establish an independent enamel-repair effect of the extract. A related in vitro study evaluated chitosan film, gel, and solution in an olive leaf extract-containing artificial saliva system using bleached enamel [28]. That substrate and those formulations differ from the present experiment, so the studies do not provide directly comparable tests of agent superiority.

The carrier response is central to the experiment. Its nominal formulation included CaCl_2_ (0.17 g/L), KH_2_PO_4_ (0.33 g/L), and Na_2_HPO_4_ (0.34 g/L), giving a combined phosphate-salt mass of 0.67 g/L before supplementation. Using the anhydrous reagent formula masses, these salt inputs correspond to approximately 1.53 mmol/L nominal calcium and 4.82 mmol/L nominal total phosphate (2.42 mmol/L from KH_2_PO_4_ and 2.40 mmol/L from Na_2_HPO_4_) before supplementation. These stoichiometric estimates are based on weighed salt inputs without batch-specific purity correction; they are not measured dissolved concentrations or free-ion activities and do not include any contribution from the extract. Soluble-ion concentrations, supersaturation, and precipitation were not measured. Carrier-associated hardness recovery is therefore compatible with mineral-containing medium exposure but cannot be attributed specifically to any carrier constituent or to olive leaf extract. Ionta et al. reported partial surface hardness recovery with artificial saliva formulations in an initial erosion model [29]; that different lesion model supports considering the medium as a contributor but does not identify the mechanism here. Importantly, within-group improvement in a supplemented formulation does not establish an incremental supplement effect when the carrier also improves. No such incremental benefit was demonstrated by the corrected between-group tests.

The major limitations constrain the principal interpretation. First, the carrier is an active calcium/phosphate-containing reference, and no ion-free or matched extract-free comparator was tested. Second, the formulations were not matched for dose or calcium/phosphate/fluoride ion availability; the low fluoride concentration may have influenced the comparison. Third, despite disclosure of the commercial extract volume loading, the absence of batch-specific phytochemical characterization and dry-matter determination limits chemically identical reproduction. Dissolved-ion measurements and stability or precipitation assessments were not performed. The 11 carrier specimens also limit precision for this central comparison. The TCP–carrier adjusted post-treatment contrast was 30.90 VHN with a multiplicity-adjusted confidence interval of −4.00 to 65.80 VHN, which includes both no difference and appreciable positive differences. Nonsignificance is not equivalence, and an incremental benefit cannot be excluded or claimed. The illustrative sensitivity threshold of d = 1.229 for 40 versus 11 specimens shows that the design is insensitive to smaller effects under that assumed model; it does not demonstrate adequate power for the actual analyses. Baseline VHN imbalance remains a limitation: supplementary ANCOVA adjusts for post-demineralization hardness only and does not directly adjust for original baseline hardness or all underlying tooth differences. Finally, prolonged static exposure differs from the oral environment [30], surface-hardness overshoot does not establish lesion repair, and the small SEM–EDX subset cannot provide confirmatory evidence.

Future experiments should use balanced, adequately planned carrier comparisons, an ion-free comparator appropriate to the model, and an otherwise matched extract-free carrier where the extract contribution is examined. Reproducible extract specification and batch characterization, dissolved calcium/phosphate/fluoride measurements, and rational dose matching are needed to distinguish agent and carrier effects. Time-course measurements, cross-sectional mineral analysis, and surface-deposit characterization should accompany pH cycling and intermittent exposure. ten Cate and Duijsters reported different mineral responses under continuous versus alternating demineralization–remineralization conditions [31], and Hannig and Balz showed protective effects of an in vivo formed salivary pellicle in an erosion model [32]. These studies support validation in more representative models, rather than clinical extrapolation from the present experiment.

## 5. Conclusions

Under continuous 28-day in vitro exposure, surface microhardness increased with both the unsupplemented calcium/phosphate-containing carrier and the supplemented formulations. At the unequal tested doses, the TCP formulation showed greater ΔVHN than the NaF and CaGP formulations, but no supplemented formulation demonstrated significantly greater surface microhardness recovery than the carrier after multiplicity correction. These formulation-specific surface responses do not establish superior remineralizing efficacy of TCP, an independent effect of olive leaf extract, or restoration of the original enamel lesion. The active carrier, small carrier group, uncharacterized batch-specific extract composition, and absence of dose or ion matching limit interpretation and reproducibility. SEM–EDX findings were descriptive and did not establish subsurface mineral restoration or the identity of a deposited mineral phase. Characterized formulations, balanced comparators, depth-sensitive measurements, and more representative exposure models are needed.

## 6. Patents

A national patent application related to the olive leaf extract-enriched artificial saliva formulation evaluated in this study is currently pending. Hülya Erten Can is the inventor and applicant.

## Figures and Tables

**Table 1:** Composition of the experimental artificial saliva formulations.

Component	NaF	TCP	CaGP	Carrier Control
KH_2_PO_4_	0.33 g/L	0.33 g/L	0.33 g/L	0.33 g/L
Na_2_HPO_4_	0.34 g/L	0.34 g/L	0.34 g/L	0.34 g/L
KCl	1.27 g/L	1.27 g/L	1.27 g/L	1.27 g/L
NaSCN	0.16 g/L	0.16 g/L	0.16 g/L	0.16 g/L
NaCl	0.58 g/L	0.58 g/L	0.58 g/L	0.58 g/L
CaCl_2_	0.17 g/L	0.17 g/L	0.17 g/L	0.17 g/L
NH_4_Cl	0.16 g/L	0.16 g/L	0.16 g/L	0.16 g/L
Urea	0.20 g/L	0.20 g/L	0.20 g/L	0.20 g/L
Glucose	0.03 g/L	0.03 g/L	0.03 g/L	0.03 g/L
Ascorbic acid	0.002 g/L	0.002 g/L	0.002 g/L	0.002 g/L
Sodium CMC	10 g/L	10 g/L	10 g/L	10 g/L
Olive leaf extract	0.5 mL/1000 mL *	0.5 mL/1000 mL *	0.5 mL/1000 mL *	0.5 mL/1000 mL *
Added agent	NaF: 3.6 mg/L	TCP: 2.48 g/L	CaGP: 2.48 g/L	—
Initial pH	6.82	6.82	6.82	6.82

* Commercial liquid olive leaf extract (Kalextract, Balıkesir, Türkiye) was added at 0.5 mL per 1000 mL of artificial saliva carrier in all four formulations (nominal loading 0.05% v/v relative to carrier volume). This volume loading does not specify dry-extract or individual phytochemical concentrations.

**Table 2:** Exploratory SEM–EDX surface elemental composition across experimental stages.

Group	Stage	Ca (wt%), Mean ± SD	P (wt%), Mean ± SD	Ca/P Ratio, Mean ± SD
NaF (*n* = 4)	Baseline	22.77 ± 10.62	11.12 ± 2.93	1.95 ± 0.51
	Post-demineralization	1.12 ± 1.92	2.30 ± 1.43	0.61 ± 1.00
	Post-treatment	18.92 ± 16.82	9.55 ± 5.16	1.66 ± 0.97
	Exact Friedman p	0.0694	0.0417	0.4306
TCP (*n* = 4)	Baseline	21.45 ± 14.19	10.90 ± 4.15	1.72 ± 0.89
	Post-demineralization	2.60 ± 4.53	4.00 ± 3.27	0.52 ± 0.52
	Post-treatment	34.42 ± 6.14	14.65 ± 2.01	2.34 ± 0.10
	Exact Friedman p	0.0046	0.0046	0.0417
CaGP (*n* = 4)	Baseline	29.50 ± 10.24	14.48 ± 4.58	2.02 ± 0.10
	Post-demineralization	9.80 ± 11.09	6.70 ± 4.51	0.98 ± 0.98
	Post-treatment	32.78 ± 8.23	14.50 ± 2.23	2.23 ± 0.27
	Exact Friedman p	0.0046	0.0417	0.0046
Carrier control (*n* = 2)	Baseline	31.65 ± 15.49	14.25 ± 5.87	2.18 ± 0.19
	Post-demineralization	0.30 ± 0.14	2.90 ± 0.85	0.12 ± 0.08
	Post-treatment	34.35 ± 13.51	15.15 ± 4.17	2.23 ± 0.28

Values are mean ± SD. Ca and P are weight percentages. Ca/P is a weight ratio, not an atomic ratio. Omnibus *p* values use exact within-specimen stage-label permutations in each supplemented group (*n* = 4). Stage-specific Wilcoxon comparisons were corrected for three contrasts per outcome; none was significant (all adjusted *p* ≥ 0.375). Omnibus findings are exploratory and not corrected across the nine elemental outcomes. Carrier-control values (*n* = 2) are descriptive.

## Data Availability

Specimen-level Vickers and SEM–EDX data are provided in the supplementary datasets (vickers analysis data.pdf and sem analysis data.pdf).

## Supplementary Materials

The following supporting information can be downloaded at: https://www.mdpi.com/article/10.3390/jfb17100528/s1. Table S1 Vickers surface microhardness, Table S2 Recovery and adjusted post treatment VHN, Table S3 Differences in mean ΔVHN, Table S4 Adjusted post treatment contrasts, Table S5 Exact SEM EDX omnibus checks, Sample size sensitivity, Supplementary datasets.

## Abbreviations

Ca	Calcium
CaGP	Calcium glycerophosphate
Ca/P	Calcium-to-phosphorus ratio
CMC	Carboxymethyl cellulose
EDX	Energy-dispersive X-ray spectroscopy
NaF	Sodium fluoride
P	Phosphorus
SEM	Scanning electron microscopy
SEM–EDX	Scanning electron microscopy coupled with energy-dispersive X-ray spectroscopy
TCP	Tricalcium phosphate
VHN	Vickers hardness number
ΔVHN	Post-treatment VHN/post-demineralization VHN

## References

[1] Dawes C., Pedersen A.L., Villa A., Ekström J., Proctor G.B., Vissink A., Aframian D., McGowan R., Aliko A., Narayana N. (2015). The Functions of Human Saliva: A Review Sponsored by the World Workshop on Oral Medicine VI. Arch. Oral Biol..

[2] Humphrey S.P., Williamson R.T. (2001). A Review of Saliva: Normal Composition, Flow, and Function. J. Prosthet. Dent..

[3] Guggenheimer J., Moore P.A. (2003). Xerostomia: Etiology, Recognition and Treatment. J. Am. Dent. Assoc..

[4] Jensen S.B., Vissink A., Limesand K.H., Reyland M.E. (2019). Salivary Gland Hypofunction and Xerostomia in Head and Neck Radiation Patients. JNCI Monogr..

[5] Assery M.K.A. (2019). Efficacy of Artificial Salivary Substitutes in Treatment of Xerostomia: A Systematic Review. J. Pharm. Bioallied Sci..

[6] Can H.E., Silkü A. (2025). Hyposalivation and Xerostomia. Salivary Analysis—Biomarkers, Clinical Applications, and Emerging Technologies.

[7] Erten Can H., Silkü Oflioğlu A., Yilmaz N.A., Altikat K.M., Yavaşer Boncooğlu R., Tuna G., Derinsu G. (2026). Antioxidant and Cytoprotective Effects of Herbal Extract-Enriched Artificial Saliva: In-Vitro Biocompatibility and Cell Culture Studies. Oral Health Prev. Dent..

[8] Grohe B., Mittler S. (2021). Advanced Non-Fluoride Approaches to Dental Enamel Remineralization: The next Level in Enamel Repair Management. Biomater. Biosyst..

[9] Featherstone J.D. (2004). Topical Effects of Fluoride in the Reversal and Prevention of Dental Decay. J. Public Health Dent..

[10] Ten Cate J. (1997). Review on Fluoride, with Special Emphasis on Calcium Fluoride Mechanisms in Caries Prevention. Eur. J. Oral Sci..

[11] Hamba H., Nakamura K., Nikaido T., Tagami J., Muramatsu T. (2020). Remineralization of Enamel Subsurface Lesions Using Toothpaste Containing Tricalcium Phosphate and Fluoride: An in Vitro µCT Analysis. BMC Oral Health.

[12] Torsakul P., Rirattanapong P., Prapansilp W. (2023). The Remineralization Effect of Calcium Glycerophosphate in Fluoride Mouth Rinse on Demineralized Primary Enamel: An in Vitro Study. J. Int. Soc. Prev. Community Dent..

[13] Šimat V., Skroza D., Tabanelli G., Čagalj M., Pasini F., Gómez-Caravaca A.M., Fernández-Fernández C., Sterniša M., Smole Možina S., Ozogul Y. (2022). Antioxidant and Antimicrobial Activity of Hydroethanolic Leaf Extracts from Six Mediterranean Olive Cultivars. Antioxidants.

[14] Mansour H.M., Zeitoun A.A., Abd-Rabou H.S., El Enshasy H.A., Dailin D.J., Zeitoun M.A., El-Sohaimy S.A. (2023). Antioxidant and Anti-Diabetic Properties of Olive (*Olea europaea*) Leaf Extracts: In Vitro and in Vivo Evaluation. Antioxidants.

[15] Krithikadatta J., Gopikrishna V., Datta M. (2014). CRIS Guidelines (Checklist for Reporting In-Vitro Studies): A Concept Note on the Need for Standardized Guidelines for Improving Quality and Transparency in Reporting In-Vitro Studies in Experimental Dental Research. J. Conserv. Dent..

[16] Aydın B., Pamir T., Baltaci A., Orman M.N., Turk T. (2015). Effect of Storage Solutions on Microhardness of Crown Enamel and Dentin. Eur. J. Dent..

[17] Aziz S., Loch C., Li K.C., Anthonappa R., Meldrum A., Ekambaram M. (2024). Remineralization Potential of Dentifrices with Calcium Sodium Phosphosilicate and Functionalized Tri-Calcium Phosphate in the Deeper Incipient Carious Lesions: An In Vitro Study. Clin. Exp. Dent. Res..

[18] Rirattanapong P., Phruksaniyom C., Asanahsark S., Cheevarungnapakul Y., Putthanuparp T., Ruangsawasdi N. (2025). Antibacterial Effects and Remineralization Potential of Silver Nanoparticles in a Novel Nanosilver Fluoride Formulation. BMC Oral Health.

[19] Montasser M.A., El-Wassefy N.A., Taha M. (2015). In Vitro Study of the Potential Protection of Sound Enamel against Demineralization. Prog. Orthod..

[20] Barrera-Ortega C.C., Rodil S.E., Silva-Bermudez P., Delgado-Cardona A., Almaguer-Flores A., Prado-Prone G. (2025). Fluoride Casein Phosphopeptide and Tri-Calcium Phosphate Treatments for Enamel Remineralization: Effects on Surface Properties and Biofilm Resistance. Dent. J..

[21] Karlinsey R.L., Mackey A.C., Walker E.R., Frederick K.E. (2010). Surfactant-Modified β-TCP: Structure, Properties, and In Vitro Remineralization of Subsurface Enamel Lesions. J. Mater. Sci. Mater. Med..

[22] Lippert F., Juthani K. (2015). Fluoride Dose-Response of Human and Bovine Enamel Artificial Caries Lesions under pH-Cycling Conditions. Clin. Oral Investig..

[23] Zaze A.C.S.F., Dias A.P., Amaral J.G., Miyasaki M.L., Sassaki K.T., Delbem A.C.B. (2014). In Situ Evaluation of Low-Fluoride Toothpastes Associated to Calcium Glycerophosphate on Enamel Remineralization. J. Dent..

[24] Thimmaiah C., Shetty P., Shetty S.B., Natarajan S., Thomas N.A. (2019). Comparative Analysis of the Remineralization Potential of CPP–ACP with Fluoride, Tri-Calcium Phosphate and Nano Hydroxyapatite Using SEM/EDX—An In Vitro Study. J. Clin. Exp. Dent..

[25] Tosco V., Vitiello F., Monterubbianesi R., Gatto M.L., Orilisi G., Mengucci P., Putignano A., Orsini G. (2023). Assessment of the Remineralizing Potential of Biomimetic Materials on Early Artificial Caries Lesions after 28 Days: An in Vitro Study. Bioengineering.

[26] Lippert F., Lynch R.J.M. (2014). Comparison of Knoop and Vickers Surface Microhardness and Transverse Microradiography for the Study of Early Caries Lesion Formation in Human and Bovine Enamel. Arch. Oral Biol..

[27] Benavente-García O., Castillo J., Lorente J., Ortuño A., Del Rio J.A. (2000). Antioxidant Activity of Phenolics Extracted from *Olea europaea* L.. Leaves. Food Chem..

[28] Silkü Oflioğlu A., Çetin D., Erten Can H., Güvenç P., Tekin İ., Altınışık H., Abdukadirov S. (2026). Comparative Remineralization Efficacy of Different Physical Forms of Chitosan in a Newly Developed Olive Leaf Extract-Containing Artificial Saliva. Molecules.

[29] Ionta F.Q., Mendonça F.L., de Oliveira G.C., de Alencar C.R.B., Honório H.M., Magalhães A.C., Rios D. (2014). In Vitro Assessment of Artificial Saliva Formulations on Initial Enamel Erosion Remineralization. J. Dent..

[30] Batista G.R., Torres C.R.G., Sener B., Attin T., Wiegand A. (2016). Artificial Saliva Formulations versus Human Saliva Pretreatment in Dental Erosion Experiments. Caries Res..

[31] ten Cate J.M., Duijsters P.P.E. (1982). Alternating Demineralization and Remineralization of Artificial Enamel Lesions. Caries Res..

[32] Hannig M., Balz M. (1999). Influence of In Vivo Formed Salivary Pellicle on Enamel Erosion. Caries Res..

