# Supplementary material for: Surface Microhardness of Enamel Exposed to Supplemented and Unsupplemented Artificial Saliva Formulations In Vitro
Source: J Funct Biomater. 2026 Oct 19;17(10):528. doi: 10.3390/jfb17100528 (PMC13705774; doi:10.3390/jfb17100528)
Supplement: Supplementary file 1 [file jfb-17-00528-s001.zip › jfb-4609865-supplementary.pdf]

Supplementary data and statistical analyses

These supplementary tables and specimen-level datasets accompany manuscript jfb-4609865. Vickers analyses include 131 independent tooth specimens. Exploratory SEM–EDX analyses include 14 different teeth measured at three stages; specimens are identified by the combination of SEM_Group and Sample_ID. Tables S1–S4 report the surface microhardness results and Table S5 reports the exploratory SEM–EDX omnibus analyses.

Table S1 Vickers surface microhardness

NaF (n = 40)

Stage	Mean ± SD	Median [Q1–Q3]
Baseline	362.85 ± 88.61	381.50 [323.50–407.75]
Post-demineralization	311.57 ± 81.00	328.00 [267.00–363.91]
Post-treatment	416.57 ± 86.03	429.33 [368.59–486.41]

TCP (n = 40)

Stage	Mean ± SD	Median [Q1–Q3]
Baseline	406.81 ± 44.99	412.67 [391.42–440.66]
Post-demineralization	330.15 ± 67.91	340.50 [305.75–383.00]
Post-treatment	468.26 ± 55.20	478.84 [442.16–493.41]

CaGP (n = 40)

Stage	Mean ± SD	Median [Q1–Q3]
Baseline	377.91 ± 64.97	381.50 [340.66–426.84]
Post-demineralization	329.23 ± 66.62	314.83 [282.59–384.59]
Post-treatment	432.04 ± 65.30	435.50 [378.92–484.59]

Carrier control (n = 11)

Stage	Mean ± SD	Median [Q1–Q3]
Baseline	352.59 ± 56.72	362.00 [316.00–392.50]
Post-demineralization	294.97 ± 66.64	302.00 [243.50–346.84]
Post-treatment	411.95 ± 57.21	413.67 [366.50–459.00]

Within-group secondary Wilcoxon tests were Bonferroni-adjusted across four groups. Adjusted $p < 0.001$ for NaF, TCP, and CaGP; adjusted $p = 0.00390625$ for the carrier control. Baseline Kruskal–Wallis $H = 12.712$, $p = 0.005302$; post-demineralization $H = 3.514$, $p = 0.318938$.

Table S2 Recovery and adjusted post treatment VHN

Group	n	ΔVHN mean ± SD	Adjusted mean [95% CI]
NaF	40	105.00 ± 53.00	423.56 [407.17–439.95]
TCP	40	138.11 ± 50.40	461.82 [448.12–475.53]
CaGP	40	102.81 ± 48.46	426.27 [412.03–440.51]
Control	11	116.98 ± 39.71	430.93 [409.57–452.28]

ΔVHN Kruskal–Wallis $H = 12.864$, $p = 0.004940$; $\varepsilon^2 = 0.078$. ANCOVA with HC3 covariance: $F(3,126) = 6.070$, $p = 0.000682$. Adjustment used the pooled post-demineralization VHN mean. Individual estimates and contrasts used the asymptotic normal reference distribution, reproducing the manuscript results.

Table S3 Differences in mean ΔVHN

Contrast	Difference	95% CI	Family adjusted CI	Adjusted p
NaF − TCP	-33.11	-55.59 to -11.08	-63.30 to -3.35	0.0272
NaF − CaGP	2.19	-19.55 to 24.12	-27.14 to 31.67	1.0000
NaF − Control	-11.98	-40.49 to 14.74	-50.86 to 23.35	1.0000
TCP − CaGP	35.30	14.18 to 56.91	6.68 to 64.66	0.0087
TCP − Control	21.13	-6.94 to 47.59	-17.36 to 56.29	0.9953
CaGP − Control	-14.16	-41.97 to 11.57	-52.27 to 19.80	1.0000

Differences are first group minus second group. Intervals are percentile bootstrap estimates using 100,000 independent specimen resamples within each group and seed 4609865. Family-adjusted intervals have individual coverage 99.1667% for six contrasts. Adjusted p values are from Mann–Whitney U tests with Bonferroni correction; these rank tests and mean-difference bootstrap intervals assess different estimands. Unadjusted rank-biserial correlations are −0.369 for NaF minus TCP and +0.414 for TCP minus CaGP.

Table S4 Adjusted post treatment contrasts

Contrast	Difference	95% CI	Family adjusted CI	Adjusted p
NaF − TCP	-38.27	-59.38 to -17.15	-66.68 to -9.85	0.0023
NaF − CaGP	-2.71	-24.14 to 18.72	-31.56 to 26.14	1.0000
NaF − Control	-7.37	-35.36 to 20.63	-45.05 to 30.32	1.0000
TCP − CaGP	35.55	16.15 to 54.96	9.43 to 61.67	0.0020
TCP − Control	30.90	4.97 to 56.83	-4.00 to 65.80	0.1171
CaGP − Control	-4.65	-30.69 to 21.38	-39.70 to 30.39	1.0000

HC3 robust Wald contrasts use the normal reference distribution. Family-adjusted intervals apply Bonferroni correction across six comparisons. For TCP versus control, the ordinary 95% interval excludes zero, but the multiplicity-adjusted interval includes zero; only the corrected comparison supports the family-level inference.

Table S5 Exact SEM EDX omnibus checks

Group	Outcome	Friedman statistic	Approximate p	Exact p
NaF	Ca	6.00	0.0498	0.0694
NaF	P	6.50	0.0388	0.0417
NaF	ratio	2.00	0.3679	0.4306
TCP	Ca	8.00	0.0183	0.0046
TCP	P	8.00	0.0183	0.0046
TCP	ratio	6.50	0.0388	0.0417
CaGP	Ca	8.00	0.0183	0.0046
CaGP	P	6.50	0.0388	0.0417
CaGP	ratio	8.00	0.0183	0.0046

Exact p values enumerate all 1296 permutations of the three stage labels within four specimens. These exploratory omnibus tests are not adjusted across nine outcomes. No Bonferroni-adjusted paired stage comparison was significant. Carrier-control inference was omitted because $n = 2$.

Sample size sensitivity

A sample-size calculation was performed before the experiment.

Sensitivity of the final sample. A sensitivity analysis was conducted using the actual group sizes, a two-sided per-comparison α of $0.05/6 = 0.00833$, and a target power of 80%. Under an illustrative normal-theory independent two-sample t-test model with equal population variances, the minimum detectable standardized mean difference was Cohen's $d = 0.796$ for 40 versus 40 specimens and $d = 1.229$ for 40 versus 11 specimens. These are sensitivity thresholds, not achieved power estimates for the Kruskal–Wallis, Mann–Whitney, or ANCOVA analyses, and do not establish that the sample was adequate for smaller effects. The SEM–EDX subset was exploratory and was not justified by this Vickers sensitivity calculation. For scale only, multiplying these thresholds by the observed pooled within-group ΔVHN SD of 49.88 gives approximately 39.70 and 61.28 VHN. This empirical SD conversion is descriptive, not a prespecified effect size or a clinically validated threshold. Computation in Python/SciPy solves for the noncentrality parameter $\delta = d / \sqrt{(1/n_1 + 1/n_2)}$, with $df = n_1 + n_2 - 2$ and the two-sided critical central t value at $\alpha = 0.00833$. Numerical integration of the normal/chi-square representation independently reproduced the quadrature calculation.

Supplementary datasets

- The accompanying files vickers analysis data.pdf and sem analysis data.pdf provide specimen-level Vickers and SEM–EDX measurements. Vickers measurements are means of the indentations at each stage. The Vickers dataset contains 131 teeth; the SEM–EDX dataset contains 42 stage records from 14 teeth. SEM specimens are identified by SEM_Group together with Sample_ID.
