# Supplementary material for: Surface Microhardness of Enamel Exposed to Supplemented and Unsupplemented Artificial Saliva Formulations In Vitro
Source: J Funct Biomater. 2026 Oct 19;17(10):528. doi: 10.3390/jfb17100528 (PMC13705774; doi:10.3390/jfb17100528)
Supplement: Supplementary file 1 [file jfb-17-00528-s001.zip › sem analysis data.pdf]

sem analysis data

Sample_ID	SEM_Group	Treatment	Stage	Point	Ca	P	Na	O	C	Al	Ca_P_ratio	key	ratio
1	Group1	NaF	Baseline	1	9.1	7.4	0.6	53.5	29.0	0.5	1.22973	Group1_1	1.2297297297297296
2	Group1	NaF	Baseline	1	20.3	10.5	0.8	44.4	23.4	0.6	1.933333	Group1_2	1.9333333333333333
3	Group1	TCP	Baseline	1	19.3	10.7	0.9	52.8	15.8	0.4	1.803738	Group1_3	1.8037383177570094
4	Group1	TCP	Baseline	1	2.2	5.1	3.1	57.9	31.4	0.4	0.431373	Group1_4	0.4313725490196079
5	Group1	CaGP	Baseline	1	30.2	14.8	0.7	46.3	7.6	0.4	2.040541	Group1_5	2.0405405405405403
6	Group1	CaGP	Baseline	1	14.8	7.9	0.9	50.0	26.0	0.5	1.873418	Group1_6	1.8734177215189873
7	Group1	Control	Baseline	1	20.7	10.1	0.7	47.2	20.7	0.5	2.049505	Group1_7	2.0495049504950495
1	Group2	NaF	Baseline	1	28.1	12.3	0.7	37.7	20.7	0.4	2.284553	Group2_1	2.2845528455284554
2	Group2	NaF	Baseline	1	33.6	14.3	0.8	41.8	9.5	0.0	2.34965	Group2_2	2.3496503496503496
3	Group2	TCP	Baseline	1	32.5	14.1	0.6	34.5	18.3	0.0	2.304965	Group2_3	2.3049645390070923
4	Group2	TCP	Baseline	1	31.8	13.7	0.8	33.6	20.0	0.0	2.321168	Group2_4	2.321167883211679
5	Group2	CaGP	Baseline	1	36.7	17.5	1.5	31.1	12.9	0.2	2.097143	Group2_5	2.0971428571428574
6	Group2	CaGP	Baseline	1	36.3	17.7	1.7	32.0	12.2	0.2	2.050847	Group2_6	2.0508474576271185
7	Group2	Control	Baseline	1	42.6	18.4	0.3	24.6	13.8	0.2	2.315217	Group2_7	2.315217391304348
1	Group1	NaF	Demin	1	4.0	1.9	0.2	48.4	42.7	2.8	2.105263	Group1_1	2.1052631578947367
2	Group1	NaF	Demin	1	0.1	0.5	1.0	54.8	37.7	0.9	0.2	Group1_2	0.2
3	Group1	TCP	Demin	1	9.4	8.4	1.0	51.5	29.1	0.6	1.119048	Group1_3	1.119047619047619
4	Group1	TCP	Demin	1	0.4	3.8	1.1	57.8	36.5	0.5	0.105263	Group1_4	0.10526315789473685
5	Group1	CaGP	Demin	1	16.8	9.4	1.1	43.6	28.5	0.6	1.787234	Group1_5	1.7872340425531914
6	Group1	CaGP	Demin	1	0.5	2.3	0.6	47.4	31.0	18.3	0.217391	Group1_6	0.2173913043478261
7	Group1	Control	Demin	1	0.4	2.3	1.2	45.7	32.0	18.4	0.173913	Group1_7	0.1739130434782609
1	Group2	NaF	Demin	1	0.2	3.8	1.0	60.4	34.2	0.4	0.052632	Group2_1	0.052631578947368425
2	Group2	NaF	Demin	1	0.2	3.0	0.9	59.7	35.7	0.5	0.066667	Group2_2	0.06666666666666667
3	Group2	TCP	Demin	1	0.4	0.5	0.1	61.9	32.3	4.7	0.8	Group2_3	0.8
4	Group2	TCP	Demin	1	0.2	3.3	1.2	61.0	34.1	0.2	0.060606	Group2_4	0.060606060606060615
5	Group2	CaGP	Demin	1	21.7	11.6	0.8	52.1	13.8	0.0	1.87069	Group2_5	1.8706896551724137
6	Group2	CaGP	Demin	1	0.2	3.5	1.2	61.6	33.1	0.4	0.057143	Group2_6	0.05714285714285715
7	Group2	Control	Demin	1	0.2	3.5	1.5	59.7	34.6	0.5	0.057143	Group2_7	0.05714285714285715
1	Group1	NaF	Remin	1	7.2	4.6	0.9	47.2	38.2	1.9	1.565217	Group1_1	1.565217391304348
2	Group1	NaF	Remin	1	1.8	5.6	0.9	55.8	35.4	0.5	0.321429	Group1_2	0.32142857142857145
3	Group1	TCP	Remin	1	33.1	14.4	0.8	39.6	11.4	0.6	2.298611	Group1_3	2.298611111111111
4	Group1	TCP	Remin	1	28.4	12.4	0.8	40.8	17.1	0.5	2.290323	Group1_4	2.290322580645161
5	Group1	CaGP	Remin	1	30.5	14.1	0.7	46.0	8.4	0.3	2.163121	Group1_5	2.1631205673758864
6	Group1	CaGP	Remin	1	22.1	11.6	0.8	55.2	10.3	0.0	1.905172	Group1_6	1.9051724137931036
7	Group1	Control	Remin	1	24.8	12.2	0.8	48.2	13.5	0.4	2.032787	Group1_7	2.0327868852459017
1	Group2	NaF	Remin	1	32.3	13.9	0.8	42.3	10.6	0.1	2.323741	Group2_1	2.3237410071942444
2	Group2	NaF	Remin	1	34.4	14.1	0.7	41.4	9.2	0.3	2.439716	Group2_2	2.4397163120567376
3	Group2	TCP	Remin	1	33.2	14.5	0.6	38.9	12.5	0.2	2.289655	Group2_3	2.2896551724137932
4	Group2	TCP	Remin	1	43.0	17.3	0.5	28.8	1.4	0.0	2.485549	Group2_4	2.485549132947977
5	Group2	CaGP	Remin	1	39.3	16.8	1.2	30.7	11.7	0.3	2.339286	Group2_5	2.339285714285714
6	Group2	CaGP	Remin	1	39.2	15.5	0.6	35.3	9.3	0.0	2.529032	Group2_6	2.5290322580645164
7	Group2	Control	Remin	1	43.9	18.1	0.3	23.7	13.8	0.2	2.425414	Group2_7	2.4254143646408837
