# Supplementary material for: Surface Microhardness of Enamel Exposed to Supplemented and Unsupplemented Artificial Saliva Formulations In Vitro
Source: J Funct Biomater. 2026 Oct 19;17(10):528. doi: 10.3390/jfb17100528 (PMC13705774; doi:10.3390/jfb17100528)
Supplement: Supplementary file 1 [file jfb-17-00528-s001.zip › vickers analysis data.pdf]

vickers analysis data

id	group	baseline	demin	post	delta
1	NaF	393.33	380.67	493.67	113.0
2	NaF	325.0	309.0	335.33	26.329999999999984
3	NaF	302.33	270.0	338.67	68.67000000000002
4	NaF	428.0	363.33	445.0	81.67000000000002
5	NaF	31.8	25.87	65.2	39.33
6	NaF	365.67	354.0	436.67	82.67000000000002
7	NaF	190.0	157.33	356.33	198.99999999999997
8	NaF	399.33	387.0	417.67	30.670000000000016
9	NaF	419.67	334.67	434.33	99.65999999999997
10	NaF	319.0	258.0	335.0	77.0
11	NaF	224.0	218.67	322.67	104.00000000000003
12	NaF	305.0	242.33	373.67	131.34
13	NaF	439.0	372.0	495.67	123.67000000000002
14	NaF	460.67	368.33	486.0	117.67000000000002
15	NaF	264.67	222.67	354.67	132.00000000000003
16	NaF	409.0	372.0	487.67	115.67000000000002
17	NaF	413.0	335.0	432.33	97.32999999999998
18	NaF	303.33	252.67	486.67	234.00000000000003
19	NaF	398.0	362.0	426.33	64.32999999999998
20	NaF	383.67	299.67	467.67	168.0
21	NaF	287.33	192.0	308.67	116.67000000000002
22	NaF	379.33	357.0	416.0	59.0
23	NaF	351.0	308.33	384.0	75.67000000000002
24	NaF	394.67	322.0	461.0	139.0
25	NaF	326.67	311.33	439.33	128.0
26	NaF	399.67	281.0	496.0	215.0
27	NaF	553.67	365.67	486.33	120.65999999999997
28	NaF	495.0	457.33	515.33	58.00000000000006
29	NaF	335.67	304.33	375.67	71.34000000000003
30	NaF	437.67	400.0	466.0	66.0
31	NaF	390.0	361.0	412.33	51.329999999999984
32	NaF	297.0	227.0	365.33	138.32999999999998

33	NaF	402.33	386.67	488.0	101.32999999999998
34	NaF	327.33	221.67	372.0	150.33
35	NaF	407.33	334.0	510.67	176.67000000000002
36	NaF	397.0	339.33	553.33	214.00000000000006
37	NaF	369.0	357.33	403.67	46.34000000000003
38	NaF	356.0	310.33	369.67	59.34000000000003
39	NaF	469.33	436.67	514.33	77.66000000000003
40	NaF	363.67	304.67	334.0	29.329999999999984
41	CaGP	305.67	255.67	402.67	147.00000000000003
42	CaGP	415.67	265.33	422.67	157.34000000000003
43	CaGP	415.67	338.67	487.33	148.65999999999997
44	CaGP	368.67	361.0	401.67	40.670000000000016
45	CaGP	377.0	312.33	503.0	190.67000000000002
46	CaGP	471.0	421.0	472.67	51.670000000000016
47	CaGP	411.33	302.67	500.67	198.0
48	CaGP	288.67	213.33	322.0	108.66999999999999
49	CaGP	440.0	415.67	471.0	55.329999999999984
50	CaGP	223.67	212.0	347.67	135.67000000000002
51	CaGP	358.33	307.67	415.0	107.32999999999998
52	CaGP	259.0	243.0	319.33	76.32999999999998
53	CaGP	405.67	362.0	480.0	118.0
54	CaGP	426.67	390.33	464.0	73.67000000000002
55	CaGP	457.67	423.67	483.67	60.0
56	CaGP	500.33	438.33	516.67	78.33999999999997
57	CaGP	434.33	419.0	528.33	109.33000000000004
58	CaGP	460.67	449.33	561.67	112.33999999999997
59	CaGP	356.33	332.0	371.0	39.0
60	CaGP	422.0	415.67	476.67	61.0
61	CaGP	344.67	293.0	441.33	148.32999999999998
62	CaGP	397.67	352.67	453.33	100.65999999999997
63	CaGP	341.33	276.0	371.67	95.67000000000002
64	CaGP	351.33	292.67	389.33	96.65999999999997
65	CaGP	325.67	290.0	370.67	80.67000000000002
66	CaGP	427.33	340.0	429.67	89.67000000000002

**67**	CaGP	302.67	290.33	349.67	59.34000000000003
**68**	CaGP	471.67	424.0	491.33	67.32999999999998
**69**	CaGP	349.33	311.33	372.67	61.34000000000003
**70**	CaGP	360.0	282.67	447.67	165.0
**71**	CaGP	338.67	282.33	381.0	98.67000000000002
**72**	CaGP	271.67	254.0	351.67	97.67000000000002
**73**	CaGP	411.0	382.67	480.67	98.0
**74**	CaGP	386.0	370.67	391.33	20.659999999999968
**75**	CaGP	350.33	317.33	386.0	68.67000000000002
**76**	CaGP	292.67	271.0	308.0	37.0
**77**	CaGP	440.67	292.33	500.0	207.67000000000002
**78**	CaGP	429.33	414.0	501.0	87.0
**79**	CaGP	307.13	230.6	403.33	172.73
**80**	CaGP	419.0	323.0	513.67	190.66999999999996
**81**	TCP	325.33	234.0	424.67	190.67000000000002
**82**	TCP	366.33	275.33	421.67	146.34000000000003
**83**	TCP	386.67	349.33	420.0	70.67000000000002
**84**	TCP	452.67	403.0	492.67	89.67000000000002
**85**	TCP	450.33	383.0	644.0	261.0
**86**	TCP	442.67	404.67	495.33	90.65999999999997
**87**	TCP	441.67	369.0	488.0	119.0
**88**	TCP	428.0	309.0	479.0	170.0
**89**	TCP	449.67	383.0	481.0	98.0
**90**	TCP	425.0	374.67	485.67	111.0
**91**	TCP	434.0	322.33	492.0	169.67000000000002
**92**	TCP	343.67	289.33	443.33	154.0
**93**	TCP	375.33	316.33	413.0	96.67000000000002
**94**	TCP	430.33	342.0	509.67	167.67000000000002
**95**	TCP	405.33	394.67	461.33	66.65999999999997
**96**	TCP	405.0	342.0	460.0	118.0
**97**	TCP	394.0	339.0	486.33	147.32999999999998
**98**	TCP	406.67	358.0	464.0	106.0
**99**	TCP	402.33	359.0	517.0	158.0
**100**	TCP	449.33	415.67	541.33	125.66000000000003

101	TCP	447.33	301.0	493.67	192.67000000000002
102	TCP	440.33	369.0	482.33	113.32999999999998
103	TCP	431.33	413.0	478.67	65.67000000000002
104	TCP	423.0	384.0	485.33	101.32999999999998
105	TCP	228.67	151.33	272.0	120.66999999999999
106	TCP	353.0	277.33	378.0	100.67000000000002
107	TCP	395.67	323.33	438.67	115.34000000000003
108	TCP	460.33	400.67	493.33	92.65999999999997
109	TCP	399.67	317.33	494.67	177.34000000000003
110	TCP	404.33	312.33	454.0	141.67000000000002
111	TCP	352.67	266.67	429.33	162.65999999999997
112	TCP	453.33	419.67	515.67	95.99999999999994
113	TCP	393.0	307.33	443.67	136.34000000000003
114	TCP	437.0	407.33	530.67	123.33999999999997
115	TCP	379.0	183.67	402.0	218.33
116	TCP	430.33	338.0	464.33	126.32999999999998
117	TCP	396.0	223.0	430.67	207.67000000000002
118	TCP	363.67	165.0	467.33	302.33
119	TCP	418.67	335.33	461.67	126.34000000000003
120	TCP	450.67	347.33	494.33	147.0
121	Control	307.0	302.0	356.67	54.670000000000016
122	Control	370.0	342.67	457.33	114.65999999999997
123	Control	271.67	201.33	413.67	212.34
124	Control	445.0	418.0	509.67	91.67000000000002
125	Control	376.33	306.0	424.33	118.32999999999998
126	Control	325.0	265.67	350.33	84.65999999999997
127	Control	409.67	351.0	466.0	115.0
128	Control	408.67	351.0	460.67	109.67000000000002
129	Control	362.0	245.67	388.0	142.33
130	Control	330.67	241.33	376.33	134.99999999999997
131	Control	272.53	220.03	328.43	108.4
